# The Internal Connection Analysis of Information Sharing and Investment Performance in the Venture Capital Network Community

**DOI:** 10.3390/ijerph182211943

**Published:** 2021-11-13

**Authors:** Bing Feng, Kaiyang Sun, Ziqi Zhong, Min Chen

**Affiliations:** 1 School of Management, Yulin University, Yulin 719000, China; zhouxiaowen@yulinu.edu.cn; 2 Department of Management, Monash University, Melbourne, VIC 3800, Australia; kaiyang.sun@monash.edu; 3 Department of Management, London School of Economics and Political Science, London WC2A 2AE, UK; z.zhong6@lse.ac.uk; 4 Academy of Financial Research, School of Business, Wenzhou University, Wenzhou 325035, China

**Keywords:** venture capital, information sharing, investment performance, capital grid, environmental-social enterprises

## Abstract

In order to explore the internal connection between information sharing and investment performance in the venture capital network community, this study took environmental-governance start-ups as the research object and used the 2009–2020 environmental-social enterprise start-up venture capital investment events as a data sample. The successful exit rate of the venture capital portfolio and the successful listing rate of investment ventures were used as the measurement criteria. Combined with regression analysis, the relationship between information sharing and investment performance in the venture capital network community was analyzed in detail. Research shows that there are differences between the ways of information sharing in the venture capital network communities. In the regression results, all coefficients are less than 0.01. There is a positive correlation between information sharing and investment performance in the venture capital network community. With the increase in enterprise characteristic variables, the degree of enterprise risk information sharing is getting higher and higher. This ultimately leads to more and more frequent corporate investment performance and a higher possibility of acquisition. Among them, the degree of information sharing in the venture capital network community is relatively high, and venture capital companies that are supported by corporate venture capital institutions will benefit even more from listed capital. Not only was the analysis of the relationship between finance and investment in the venture capital network community pointed out in this research, but also the investment development of entrepreneurial enterprises was also provided with feasible suggestions.

## 1. Introduction

Venture capital is a form of capital with high risk and high income. Venture capital mostly uses private equity funds to gain market recognition for related companies. Venture capital is often utilized by small- and medium-sized enterprises with high growth and high risk. These companies are short of funds for early development, but the technology and capabilities of these companies can often bring huge benefits to the capital market [1]. This is different from other private equity funds, but investments also face problems such as the need to burn a lot of money or return to this cycle for a longer period of time. The main reason for this situation is that these innovative SMEs often have certain differences in enterprise management and market expectations, coupled with the uncertainty of the development of these start-ups, which greatly affect the capital investment of these enterprises [2]. Some scholars have shown that more than 75% of the venture capital enterprises from the middle of the 20th century will result in corporate bankruptcy. For the other 25% of the remaining investment events, these start-ups will often use the method of listing or being acquired in order to rapidly increase their original capital accumulation, which causes venture capital enterprises to obtain a large amount of considerable benefits in a short time [3]. Among them, the most typical example is the listing of Shun Net Technology on the GEM, causing its original capital to surge from 1 to 215, which is highly recognized by the capital market for relevant enterprises [4]. China Youth Tourism benefited from 8.4 times its original capital by acquiring Wuzhen Tourism. Jumei Goods was also listed on the NASDAQ Main Board through venture capital, which ultimately caused its original capital to increase by more than 600% [5]. However, as China has paid more attention to scientific and technological innovation in recent years, many universities and enterprises have also increased R&D costs. Because innovation and entrepreneurship market information disclosures are not timely, many risk institutions in the investment process often face incomplete information, making decision making more difficult [6]. Therefore, in order to effectively solve these problems, it is necessary to deeply understand the relationship between information sharing and investment performance in the venture capital network community, which has important reference value for promoting the development of venture capital market and the national enterprise innovation industry.

Information sharing refers to the use of information systems and related communication tools among different departments, industries, and enterprises in order to transfer social information, integrate social resources, save social costs, and create more value [7]. Venture capital performance refers to the benefits of venture capital institutions through investment in enterprises over a certain period of time and under a specific market structure. These factors also include the overall social macro situation [8]. Although the relevant venture capital institutions can better understand and analyze the relevant data, the existing venture capital institutions, in order to obtain more information, will reduce investment risk to achieve cross-industry venture capital. This greatly affects the existing industrial development, but also causes the members of the entire investment market to be in series with each other, and ultimately builds a risk capital network community [9]. Enterprises in the community tend to help these SMEs to quickly gain capital attention through information sharing, to a certain extent, and many enterprises usually have excessive packaging, which greatly affects the development of normal enterprises and capital markets [10]. Using existing tools to correctly identify and analyze these companies, and to understand the relationship between corporate information sharing and investment performance, will help start-up companies find appropriate positioning and development goals. This can provide feasible suggestions for more venture capital companies. Therefore, this study has important reference value for promoting the venture capital industry and the national industrial innovation.

On this basis, this study expects to clarify the relationship between information sharing and investment performance in the venture capital network community through the analysis of relevant enterprise data. This study takes environmental-social governance start-ups as the research object, puts forward reasonable assumptions with the help of relevant references, uses the network analysis to analyze in the dimension of network community research, and implements the relevant venture capital network model. The effectiveness of the model proposed here is further verified by data tests and empirical analysis. Section 1 is the theory introduction, and Section 2 is the method introduction, hypothesis, and construction of the model of information sharing and investment performance in venture capital. Meanwhile, Section 2 conducts empirical research by the venture capital data of enterprises from 2009 to 2020. Finally, Section 3 analyzes the results and verifies the hypothesis. This study has important reference significance for the development of national innovation and entrepreneurship industries.

## 2. Research Methods and Design

### 2.1. Network Community Research

The network community is the sum of the social network analyses that are formed between different social subjects, which contain the content of correlation matrix and graph theory. Through the weight between different communities, the network community relationship of related enterprises is finally built. This study adopts the method of network community research, which involves the analysis of the relationship among social subjects, subjects, and actors in the network, and includes the resources and information network that is built between the subjects of the network community. Although society has a certain degree of stability, other people in the online community will also be subject to corresponding restrictions. This will reduce the communication of people in the online community and further reduce the exchange of information [11,12].

Degree centrality [13], eigenvector centrality [14], intermediate centrality [15] and closeness centrality [16] are used as metrics. Among them, the degree centrality is used to calculate the number of relationships between all of the venture capital institutions and the other connections in the existing network community. The specific calculation process is as follows:

(1) $$d_{i}=\sum_{j\neq i}X_{ij}$$

In (1), i denotes different venture capital enterprises with characteristics, and j denotes odd venture capital enterprises after *i* is removed from the network community.$X_{ij}$ represents the relationship between venture capital institutions and investment performance in a specific trading year for five years. If investment is added, it is 1; otherwise, it is 0. The current degree center index is the basic index for calculating venture capital enterprises, and the more that relevant enterprises invest, the more information and resources the investment institutions obtain. However, due to the characteristics of gridding, the calculation index of the degree center directly leads to the difference of the degree center only when it compares the same social network or a network community of the same scale. Therefore, it is difficult to analyze the core changes in different scales of network communities. Hence, to further eliminate the differences that are caused by the scale of network communities [17], this study standardizes it and gets the relative degree centrality. The specific calculation is as follows:

(2) $$d_{i}{}^{\prime }=\frac{1}{N-1}\sum_{j\neq i}X_{ij}=\frac{d_{i}}{N-1}$$

Since the degree centrality is only a simple analysis index, and relevant scholars have proposed the eigenvector centrality index, this index analyzes the importance of other venture capital institutions that are connected with venture capital institutions in the network. By cooperating with other venture capital institutions that have a higher centrality, the centrality of the feature vectors of the institutions will also increase accordingly. The eigenvector centrality of venture capital institutions can be expressed as:

(3) $$e_{i}=\lambda \sum_{j=1}^{N}X_{i,j}e_{j}$$

λ is a constant, representing the maximum eigenvalue of the matrix and the centrality of the eigenvector, and $e_{j}$ is the situation of venture capital institutions and joint investment. The data are standardized, and the index of intermediate centrality mainly refers to the relationship between two venture capital enterprises without any cooperation opportunities, in which the intermediary plays an important role. It mainly depends on more intermediate institutions to build the relationship between all of the subjects, and is calculated as Equation (4).

(4) $$b_{i}=\sum_{j<k}\frac{p_{ijk}}{p_{jk}}$$

$p_{jk}$ represents the total number of shortest paths connecting venture capital institutions to *j* and *k*, and $p_{ijk}$ represents the number of paths connecting venture capital institutions to *j* and *k* through institution *i*. If venture capital needs to ensure a certain degree of real time, then it requires better network accessibility, which requires only a short time to obtain effective network information data and will involve the connection distance. The specific calculation is as follows:

(5) $$c_{i}=\frac{N-1}{\sum_{j\neq i}dis(i,j)}$$

dis(i,j) represents the shortest step connecting venture capital institutions *i* and *j* in the network. If venture capital *i* and *j* are connected at the shortest distance through two “intermediary” institutions, the shortest step number between *i* and *j* is 3. If venture capital institutions *i* and *j* relate to each other due to joint investment behavior, the shortest step number of connecting institutions is 1.

### 2.2. Research Hypotheses and Data

Many scholars have studied the relationship between the network community of venture capital institutions and the investment portfolio. Among them, Abdulla et al. (2021) implemented a structural equation model to explain the complex relationship between the social network and corporate performance by introducing the intermediary roles of trust, sales ability, and pricing ability. Then, using the empirical data of 380 SMEs in Indonesia, they found that the use of social media in the management process did not affect the growing corporate performance. Trusted social networks enable enterprises to obtain pricing and sales capabilities, which have a positive impact on corporate performance [18]. Tatiana and Magdalena (2020) conducted nine semi-structured interviews in order to examine the role of relationships in creating and maintaining portfolios by using the four stages of the relationship marketing process model. It was found that long-term funding contracts were used as a mechanism for establishing relationships and were the driving force for interactive investment. Network communities also establish relationships with capital investors by managing the relationships between target stakeholders and activities [19]. Jan and Vlachopoulos (2019) used social network analysis to identify communities in online learning of higher education and studied their problems. The results proved the value of community-based learning. Although, in the traditional, mixed or online environment, the practice community and inquiry community are still perfect and empirically tested frameworks, which have been effectively used to explore community-based learning in professional and educational environments [20]. Bolivar et al. (2021) tested how the access and mobilization of network resources affects the performance of the company and examined the extent to which the level of network resource mobilization of the company plays a role in improving financial performance. The level of network resource mobilization has an inverted U-shaped relationship with income performance, thus revealing the limitations and considerations of strategic alliance strategy [21]. According to the above research, there is an important relationship between the network community and the portfolio, so the relevant assumptions are proposed:

**Hypothesis** **1** **(H1).** *The network community of venture capital institutions is conducive to improving the successful exit rate of portfolio*.

**Hypothesis** **2** **(H2).** *Venture capital institutions’ network community is conducive to the successful listing or acquisition*.

Many scholars have also reported the relationship between the degree of information sharing and the Initial Public Offering (IPO) of an enterprise. Among them, Hu et al. (2020), using the dynamic game model of real options, studied the relationship between pricing constraints and IPO timing complexity in the stock market, and found that IPO timing was a complex dynamic game in the stock market. The model showed that IPO pricing constraints reduced the exercise value of real options at the IPO timing, thus limiting the independent timing of enterprises and promoting early listing. The IPO price limit had a great influence on high-quality enterprises such as science and technology enterprises [22]. Wu et al. (2020) studied the impact of network structure on the successful exit of venture capital alliances from emerging markets through the empirical analysis of China’s joint VC data. Compared with mature capital markets, this mechanism not only has some commonalities, but also shows the particularity of emerging markets. In general, the mechanism has a positive effect on successful exit by obtaining heterogeneous information [23]. Using the data of the GEM-listed companies from 2009 to 2018, Guo et al. (2021) empirically studied the relationship among R & D investment, venture capital (VC) syndicates, and IPO underpricing. They found that there was a significant positive correlation between R & D investment and IPO underpricing, indicating that the higher the R & D investment, the higher the degree of IPO underpricing. VC joint intervention played an “adverse selection” role, did not play the advantages of information sharing, and exacerbated the positive correlation between R & D investment and IPO underpricing [24]. From the above research, the relevant research hypotheses are proposed:

**Hypothesis** **3a** **(H3a).** *The degree of information sharing of venture capital institutions is positively correlated with the first day earnings of IPO*.

**Hypothesis** **3b** **(H3b).** *The degree of information sharing of venture capital institutions is negatively correlated with the first day return of the IPO of start-ups*.

**Figure 1:**
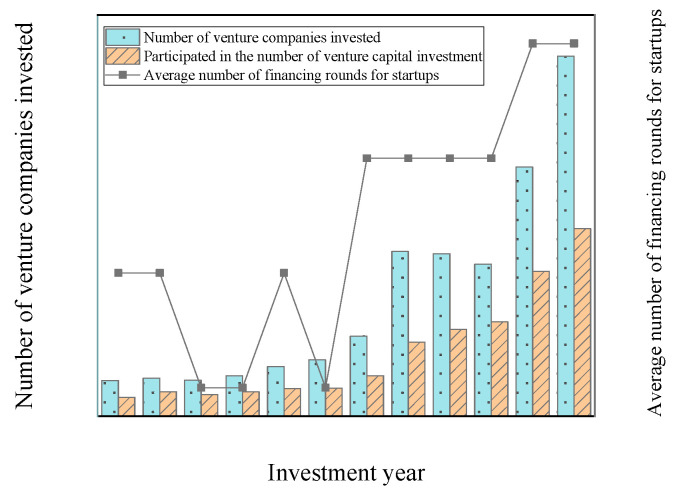
Venture capital transactions from 2009 to 2020.

Research data mainly comes from the CV database (https://data.cvsource.com.cn/cn/login (accessed on 12 June 2015)). The database contains all venture capital transactions from 1989 to 2020, mainly involving investment institutions, investment information, investment amounts, transaction information, innovation and entrepreneurship information, and the overall evaluation level of the industry. Risk investment transaction events are accompanied by detailed data content [25]. The first day of IPO transactions and other financial information data of listed companies come from the CSMAR (https://us.gtadata.com/ (accessed on 12 June 2015)) database [26]. Figure 1 shows the risk investment information for some years. The data used in Figure 1 are corporate venture capital data from 2009 to 2020. The data source is the CV database (https://data.cvsource.com.cn/cn/login (accessed on 12 June 2015).). The research objects in Figure 1 are environmental-social enterprises and start-up companies.

As the information Figure 1 suggests, since 2009, China’s venture capital transaction projects have been expanding rapidly. Among them, the total number of invested enterprises has increased rapidly. Compared with 2019, the number of enterprises in 2020 has increased by 30.78%, and the number of venture capital investments has also increased by 22.86%. The number of rounds of average financing of start-ups is increasing, but it also shows a trend of volatility growth, which indicates that venture capital enterprises have an important relationship with investment performance.

### 2.3. Implementation of Research Model

In order to further determine the relationship between information sharing and investment performance in venture capital that is proposed here, the following model was implemented [27]. The specific calculation is as follows:

(6) $$Exit\;rate_{i,t}=\alpha +\beta \times centrality_{i,t}+\sum_{j}\gamma_{j}\times controls_{j,t}+\lambda_{k}+\varepsilon_{i,t}$$

The proportion of successful venture capital firms launched by $Exit\;rate_{i,t}$ is also a proportion of all of the venture capital firms that were acquired through IPOs. $d_{i,t}$ is the degree interpretation center, $e_{i,t}$ is the characteristic vector $\lambda_{k}$ centrality, $b_{i,t}$ is the intermediate centrality, $c_{i,t}$ is the closeness centrality, $controls_{j,t}$ is the fixed variable affecting the performance of venture capital, $\sum_{j}\gamma_{j}$ is the effect factor that is difficult to determine, such as the industry year, and $\varepsilon_{i,t}$ is the change term. α,β is the fixed coefficient. To further determine whether the venture capital network society will affect the capital realization of investment-related enterprises after listing, this study uses the following model for regression analysis:

(7) $$Exit_{i}=\alpha +\beta \times centrality_{i}+\sum_{j}\gamma_{j}\times controls_{j}+\lambda_{k}+\varepsilon_{i}$$

$Exit\;rate_{i}$ is the virtual variable for the successful introduction of venture capital institutions, and the successful merger is 1, otherwise it is 0. $d_{i}$ is the degree interpretation center, $e_{i}$ is the characteristic vector $\lambda_{k}$ centrality, $b_{i}$ is the intermediate centrality, $c_{i}$ is the closeness centrality, $controls_{j}$ is the fixed variable affecting the performance of venture capital, $\sum_{j}\gamma_{j}$ is the effect factor that is difficult to determine, such as the industry year, $\varepsilon_{i}$ is the change term, and α,β is the fixed coefficient. In order to further analyze the relationship between information sharing and corporate IPO, the model that was implemented is expressed as (8):

(8) $$IPOreturn_{i}=\alpha +\beta \times centrality_{i}+\sum_{j}\gamma_{j}\times controls_{j}+\lambda_{k}+\varepsilon_{i}$$

$IPOreturn_{i}$ is the gap between the closing price and the issuing price of the first day after the listing of innovative enterprises. $centrality_{i}$ is the degree of information sharing of the relevant investment institutions in the network community, and $controls_{j}$ is the control variable in the first day of the IPO.

### 2.4. Definition of Research Variables

The explained variable is the venture capital performance (successful exit rate of portfolio); since many innovative enterprises will be liquidated due to a lack of experience and related management problems, such venture capital often needs higher investment returns. Therefore, many scholars have made the relevant measurements. Enrica et al. (2019) studied the relationship between external and internal factors and SPM adoption by using new data sets from 1864 emerging social enterprises around the world. The results showed that the adoption of venture capital performance in social enterprises was related to the successful exit rate of the portfolio [28]. Sun et al. (2020) analyzed the impact of venture capital on innovation performance. The number of patent applications and the patent quality were used as variables, and the data showed that innovation performance is significantly improved by venture capital. For industries with a high dependence on foreign financing, a high technical intensity, and areas with better property rights protection, venture capital has a more significant role in promoting innovation performance [29]. Zhang et al. (2021) used regression to examine the causes and consequences of herding among venture capitalists. The results showed that herd behavior in the venture capital market was driven by positive signals of basic information and a higher degree of information uncertainty [30]. The final calculation method is as follows:

(9) $$m_{i}=m_{x}/m_{y}$$

$m_{x}$ is the number of successful exit enterprises, $m_{y}$ is the number of investment enterprises, and $m_{i}$ is the investment performance of venture capital. The average value before December 31, 2020 in successful listing or M & A is set to be 1; otherwise, it is 0. The IPO earnings of innovative enterprises on the first trading day are also used as a performance measure in many literature sources. The difference between closing price and issuing price is calculated as follows:

(10) $$IPOreturn=\frac{p_{1}-p_{0}}{p_{0}}$$

$p_{1}$ is the closing price, $p_{0}$ is the issuance price, and IPOreturn is the difference between the two. The key explanatory variables are: $d_{i}$ as the degree explanatory center, $e_{i}$ as the characteristic vector $\lambda_{k}$ centrality, $b_{i}$ as the intermediate centrality, and $c_{i}$ as the closeness centrality. For the control variables, the number of venture capital institutions, the sources of funding and institutional reputation, the overall investment environment, the joint investment, the financing scale, the initial investment, the start-up industry, and other information from successful listed start-ups were used.

## 3. Research Results and Analysis

### 3.1. Descriptive Statistics

**Figure 2:**
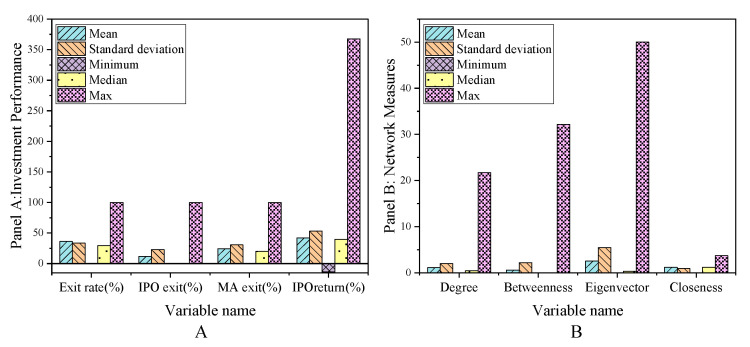
Descriptive statistical results of the main variables in the sample interval (**A**) Panel A; (**B**) Panel B.

**Figure 3:**
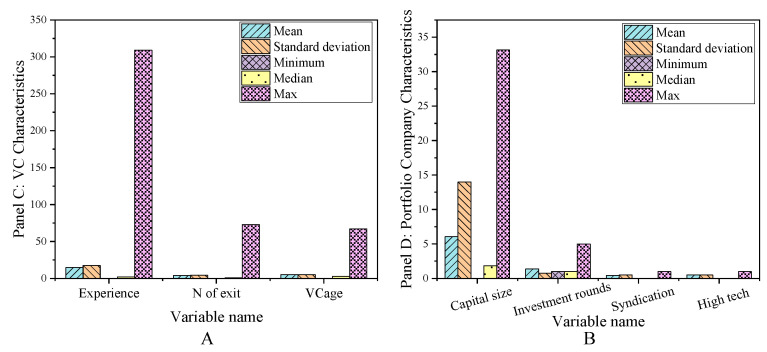
Descriptive statistical results of main variables in sample interval (**A**) Panel C; (**B**) Panel D.

**Figure 4:**
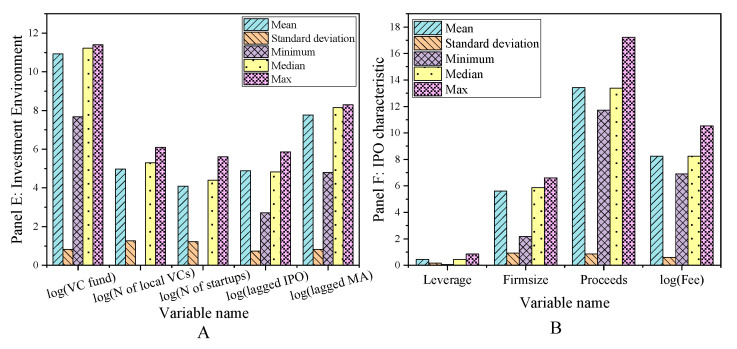
Descriptive statistical results of main variables in sample interval (**A**) Panel E; (**B**) Panel F.

The main variables of the sample interval are exit, network centrality, other characteristics of venture capital institutions, entrepreneurial enterprise characteristics, venture capital market environment, and listed enterprise characteristics. The descriptive statistical results are shown in Figure 2, Figure 3 and Figure 4.

**Figure 5:**
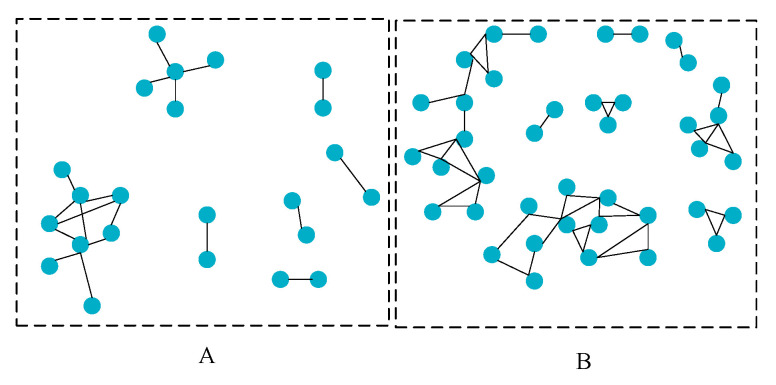
Social network of venture capital (**A**) 2009; (**B**) 2010.

Panel A shows that the average successful exit rate of venture capital from 2000 to 2011 is 36.15%, over which time the IPO exit rate is 11.68% and M & A exit rate is 24.47%. Figure 2 shows the social network centrality index of venture capital institutions. Among them, the average value of the centrality of venture capital institutions is 1.08%. This means that on average, each venture capital institution has a joint investment relationship with nearly 1.08% of the other venture capital institutions in the network. This conclusion is basically consistent with the conclusion that was drawn by Glaziev (2021), namely that a small number of venture capital institutions have joint investments. The relationship between co-invested venture capital institutions is quantified here [31]. The path through a specific venture capital institution accounts for about 0.57%, and the reciprocal of the distance to the sum of other venture capital institutions in the network is 1.18% on average. The social network of venture capital institutions in 2009 and 2010 is shown in Figure 5.

**Figure 6:**
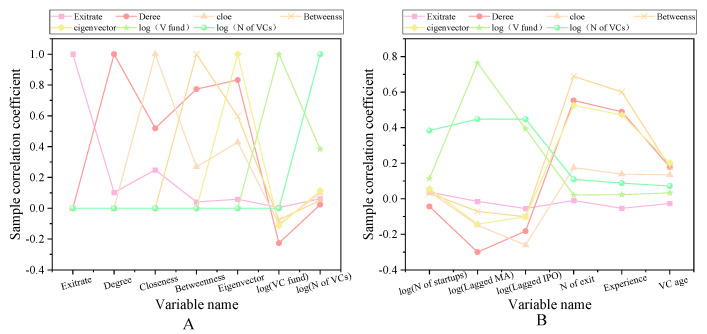
Correlation coefficient diagram of main variables (**A**) First seven variables. (**B**) Other variables.

Figure 5A shows that there are only 23 nodes in the network structure, indicating that only 23 venture capital institutions have joint investment relationships. In 2010, the number of nodes in the network structure diagram increased to 40. For most venture capital institutions, they only have a joint investment relationship with a few institutions, and a few venture capital institutions hold an absolute advantage in the network, in order to maintain a joint investment relationship. The correlation coefficient of the main variables is shown in Figure 6.

In Figure 6, there is a significant positive correlation between the four network centrality indicators and the successful exit rate of the venture portfolio. The correlation coefficients of the degree centrality index and close centrality, intermediate centrality and eigenvector centrality are significantly higher, exceeding 50%. The correlation coefficient between the proximity centrality index and the other three centrality indexes is less than 50%, indicating that the four indexes contain the same information and have some differences. This part of the result is basically the same as the previous research results of Hasheminejad and Pishvaee (2021), that is, that the successful launch rate of the venture portfolio is related to the network center. The centrality of these networks is refined here, and the specific centrality coefficients are obtained [32].

### 3.2. Data Analysis Results

**Figure 7:**
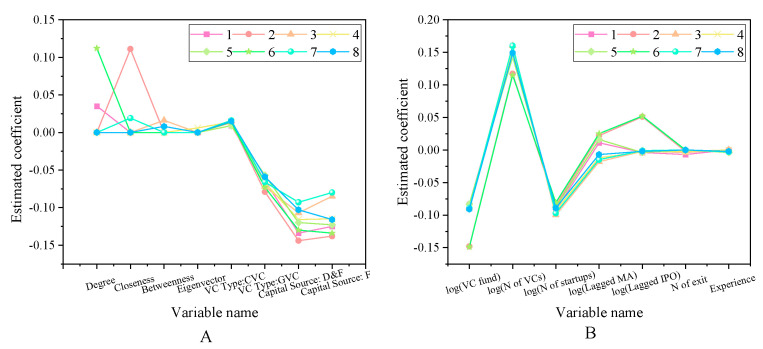
The impact of venture capital social network on successful exit rate (**A**) The first eight variables. (**B**) Other variables.

The impact of the venture capital social network on successful exit rate is shown in Figure 7.

**Figure 8:**
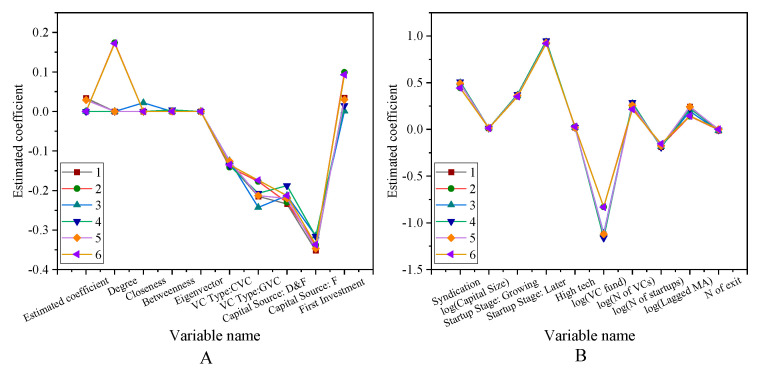
Impact of venture capital social networks on entrepreneurial performance (**A**) Top 10 variables (**B**) Other variables.

After controlling the market investment environment and other characteristics of venture capital institutions, the regression coefficients of the social network centrality index of venture capital institutions are significantly positive, so Hypothesis 1 is valid. The social network is beneficial to improve the value-added service ability of venture capital institutions, so it can play a positive role in the successful exit of entrepreneurial enterprises in the portfolio. Pandher (2021) pointed out that the exit of start-ups is related to the investment portfolio. On this basis, the social network has a positive effect on the value-added service capabilities of venture capital institutions [33]. The impact of venture capital social networks on entrepreneurial performance is shown in Figure 8.

**Figure 9:**
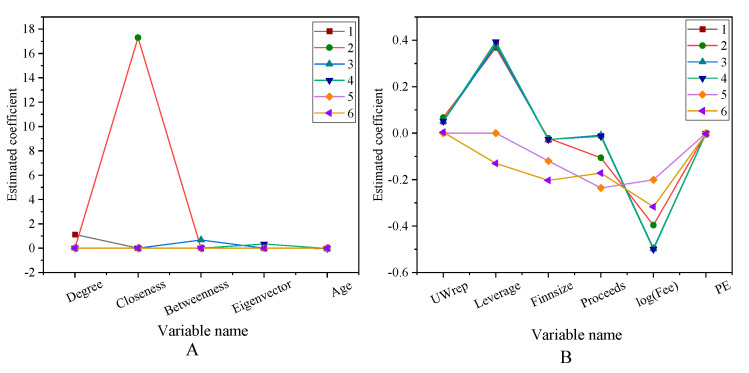
The impact of venture capital social network on IPO first-day earnings (**A**) First five variables. (**B**) Other variables.

According to the results of Figure 8, after controlling the other characteristics of venture capital institutions, entrepreneurial characteristics and market investment environment and other factors, the social network centrality of venture capital institutions has a significant positive explanatory power for the successful listing or mergers and acquisitions of entrepreneurial enterprises. This is basically consistent with the results of Scarlata et al. (2021) regarding the listing of venture capital institutions and start-ups. But here we have a study on the centrality of social networks [34], which confirms Hypothesis 2. The impact of venture capital social network on IPO first-day earnings is shown in Figure 9.

In Figure 9, from the above three indicators, there is a large fluctuation in closeness, and there is also a large fluctuation in Figure 9B, which proves the effectiveness of Hypothesis 3 proposed here. When a large amount of venture capital is invested in the network community, the investment performance will be continuously improved. This is consistent with the research results of online communities and investment performance that was obtained by Duening et al. (2021) [35].

### 3.3. Depth Analysis Test

**Figure 10:**
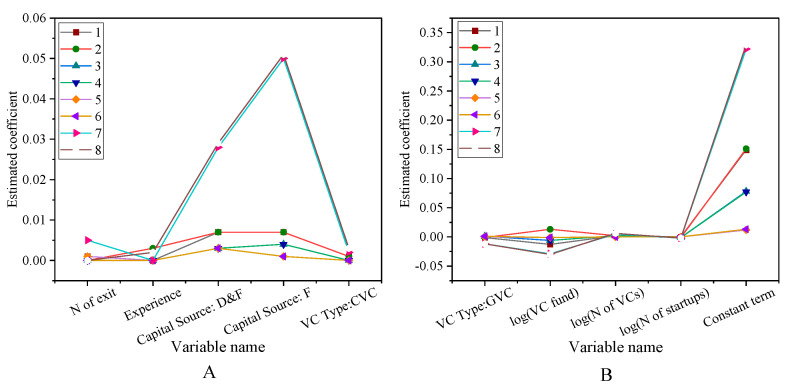
Test results of risk investment social network centrality factors (**A**) The first five variables. (**B**) Other variables.

Figure 10 presents the risk investment social network centrality test results.

**Figure 11:**
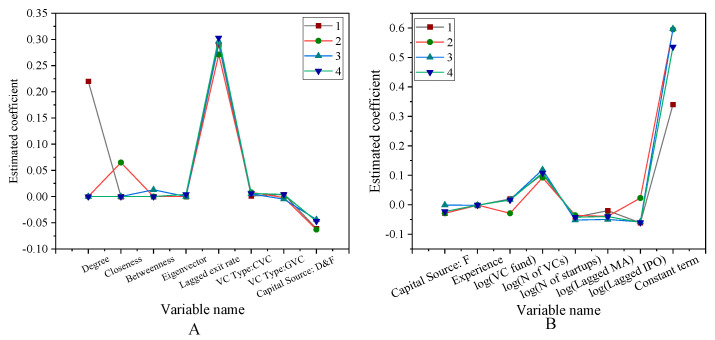
Results of the performance persistence test for venture capital firms (**A**) The first eight variables. (**B**) Other variables.

The results show that the Nofexit and Experience coefficients are significantly positive, indicating that the reputation of venture capital institutions helps to improve the network centrality. Compared with venture capital institutions with a Chinese background, venture capital institutions with foreign capital and Chinese-foreign joint ventures have richer investment experience and more professional investment skills. Thus, their social network centralities are significantly higher. The persistence test of venture capital institutions is shown in Figure 11.

In Figure 11, the horizontal coordinates are different venture capital institutions, and the vertical coordinates are the results of the persistence test. Among them, Figure 11A shows that there are significant differences in the information sharing modes in the venture capital network community. In the regression results, all of the coefficients are less than 0.01. This shows that there are very significant differences among these indicators. After the historical exit rate is fixed, the coefficients of the existing institutional venture capital and the network community in the institutions are positive. It also shows that some parts of the network community may be involved in information sharing, that is, the information sharing mechanism of some enterprises promotes the development of related enterprises. Nahlik and Fabozzi (2021) have shown that information sharing can promote the development of enterprises. These promotion indicators are quantified here [36].

### 3.4. Robustness Test

**Figure 12:**
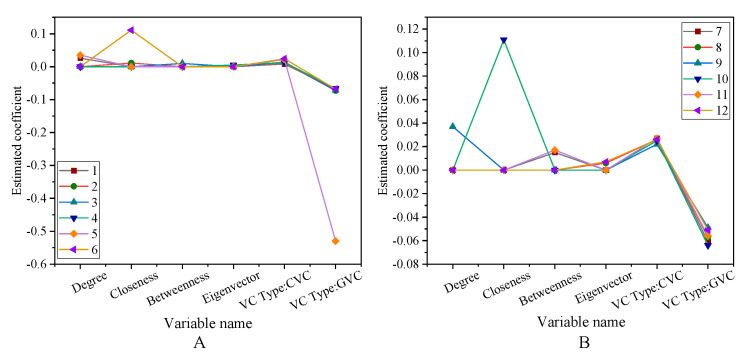
Robustness test for portfolio successful exit rates (**A**) The first six variables 1–6. (**B**) The first six variables 7–12.

**Figure 13:**
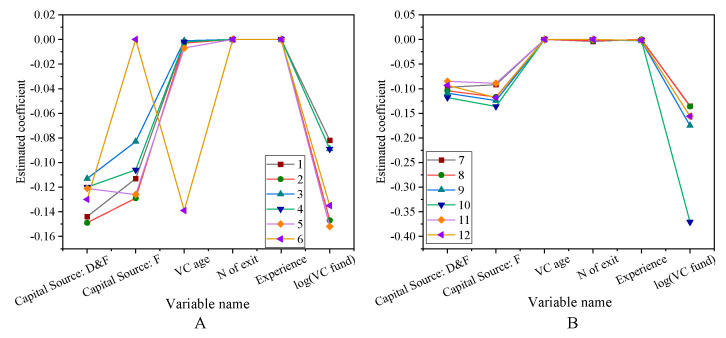
Robustness test for portfolio successful exit rates (**A**) Other variables 1–6; (**B**) Other variables 7–12.

The robustness test of the portfolio’s successful exit rate is shown in Figure 12 and Figure 13.

**Figure 14:**
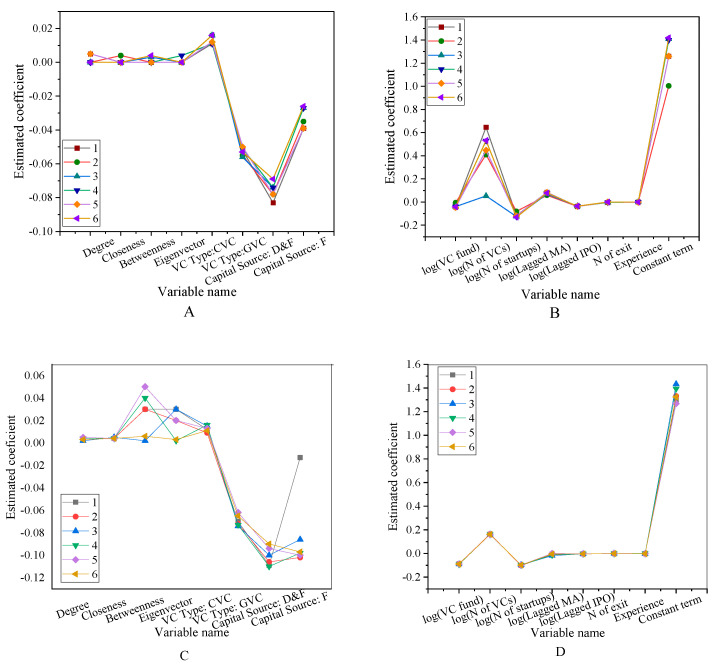
Robustness test of successful exit rate of social network and portfolio on region and category (**A**,**C**) are the first 8 variables; (**B**,**D**) are other variables.

The robustness test is to test the robustness of the successful exit rate of the portfolio. Among them, Figure 12A is the test of the first 6 variables 1–6 items, and Figure 12B is the test of the first 6 variables 7–12 items. Figure 13A is the test result of 1–6 items of other variables, and Figure 13B is the test result of 7–12 items of other variables. The test results show that the significance of all the variables fluctuates between −0.4–0.1. Among them, most of them are concentrated between −0.1–0.1. Using different exit window times or other indicators of reputation agents, the results show significant robustness. The robustness test of the region’s social network and the successful exit rate of the investment portfolio is shown in Figure 14.

In Figure 14, as the address of the venture capital network community is constantly changing, the headquarters area and different venture capital analogies have a significant impact on the investment portfolio. The change of the valuation coefficient is between −0.2 and 1.6, indicating that the centrality of the social network of venture capital institutions is beneficial to increase the successful launch rate of the investment portfolio. This result is basically consistent with the research results of Koenig and Burghof (2021) on the relationship between venture capital institutions and portfolio launch rates. On this basis, it is concluded that the factor that affects the successful launch rate of the investment portfolio is the centrality of the social network [37]. In the end, the assumption that was made in the previous article holds true here.

## 4. Discussion

At present, in terms of the background of venture capital institutions, joint investment and launch environment, factors for the successful exit of Chinese venture capital institutions are being discussed. Taking the social network of venture capital institutions as a research perspective, this study systematically analyzes the impact of venture capital institutions’ social networks on investment performance through empirical research on the venture capital transactions of venture capital institutions that were headquartered in mainland China from 2009 to 2020. After controlling the influence of factors such as the characteristics of venture capital institutions and the market investment environment, the social network of venture capital institutions has a significant positive impact on the success of the investment portfolio. After controlling the characteristics of the invested ventures, there is also a significant positive relationship between the social network of venture capital institutions and the successful listing or merger of ventures. From the two aspects of the portfolio investment performance of venture capital institutions and the successful performance of start-up enterprises, the positive role of the social network of venture capital institutions in China’s venture capital field is confirmed. Secondly, the research on the first-day earnings of start-ups found that venture capital institutions with higher network centers are more likely to be recognized by the market. Therefore, the listing of venture companies that are participating in the investment can better attract investors to subscribe in the secondary market and push up the return on the first day of listing. Through the research on the factors that influence the social network position of the venture capital institution, the positive influence relationship between the historical investment behavior of the venture capital institution and the centrality of the social network is confirmed. Venture capital institutions with a richer investment experience and outstanding investment performance are more likely to actively invite or passively be invited to become joint investment targets.

## 5. Conclusions

In order to effectively analyze the problems of venture capital and reduce the problems that result in difficult decision making due to the non-disclosure of information, the related theories of venture capital were used, taking venture capital-related online communities as the research object and using corporate venture capital data from 2009 to 2020 as a model. The centrality of the online community was used as a basic indicator, which extends a variety of calculation methods. According to the summary of the literature, relevant research hypotheses and their corresponding analysis models were constructed. Finally, the effectiveness of the proposed model was further verified through empirical data. Among them, there was a positive correlation between information sharing and investment performance in venture capital network communities. When the value of enterprise characteristic variables continues to increase, the degree of enterprise risk information sharing becomes higher and higher, which causes a higher possibility of the enterprise investment performance to accelerate acquisition. The higher the degree of sharing in the online community, the higher the capital benefit that will be determined. This has important reference value for promoting the research of venture capital-related fields and provides a direction for the development of enterprise performance in the field of venture capital. Meanwhile, this study provides actionable points for the listing of joint venture capital companies, namely, adjusting the social network centrality of venture capital institutions, and it has certain practical significance. Although, the relationship between corporate information sharing and investment performance was resolved. The disadvantages are firstly, only selected environmental-social enterprise start-ups were analyzed. The sample was unitary and may have had certain limitations for other related companies. Therefore, the sample needs to be further expanded. Secondly, it was confirmed that information sharing in online communities is crucial to investment performance; there is a positive correlation between them. However, this conclusion cannot effectively identify the influence channels of online communities. After that, the possible mediating effect between the two needs to be analyzed. Meanwhile, the sample needs to be continuously expanded, and more in-depth mechanisms need to be further analyzed. The research content will be mainly applied in the field of venture capital development.

## Data Availability

The raw data supporting the conclusions of this article will be made available by the authors, without undue reservation.
